# The 5 R’s of Indigenous Research as a Framework to Co-Design and Evaluate an Outdoor Play Program in Early Learning and Child Care Centers: Protocol for the Promoting Early Childhood Outside (PRO-ECO) 2.0 Wait-List Control Cluster Randomized Trial

**DOI:** 10.2196/77956

**Published:** 2025-12-12

**Authors:** Mariana Brussoni, Rachel Ramsden, Sheila Grieve, Dawn Mount, Emily Fox, Susan Herrington, Yingyi Lin, Stella Erasmus Johnson, Jean Lloyd, Enid Elliot, Emily Mlieczko, Andrea Lemire, Laranna Scott, Ashley Barrett, Emma Cottier, Ally Rice

**Affiliations:** 1 Department of Pediatrics University of British Columbia Vancouver, BC Canada; 2 British Columbia Children's Hospital Research Institute Vancouver, BC Canada; 3 Human Early Learning Partnership University of British Columbia Vancouver Canada; 4 School of Population and Public Health University of British Columbia Vancouver, BC Canada; 5 Vancouver Island University Nanaimo, BC Canada; 6 School of Architecture and Landscape Architecture University of British Columbia Vancouver, BC Canada; 7 Independent Consultant Seattle, WA United States; 8 Knowledge Keeper Nanaimo, BC Canada; 9 Knowledge Keeper Christina Lake, BC Canada; 10 PRO-ECO 2.0 project Victoria, BC Canada; 11 PRO-ECO 2.0 project Nanaimo, BC Canada; 12 PRO-ECO 2.0 project Vancouver, BC Canada; 13 PRO-ECO 2.0 project Grand Forks, BC Canada; 14 PRO-ECO 2.0 project Moberly Lake, BC Canada; 15 PRO-ECO 2.0 project Cranbrook, BC Canada

**Keywords:** early childhood education and care, indigenous data sovereignty, risky play, community-based research, methodology

## Abstract

**Background:**

Outdoor play is a fundamental part of childhood. Children’s participation in outdoor play connects them to nature and the land, and supports their role in the natural world. Early learning and child care (ELCC) centers provide opportunities for outdoor play; however, barriers toward the provision of outdoor play exist, including educator attitudes, policies and procedures, outdoor space limitations, and adverse weather conditions.

**Objective:**

The Promoting Early Childhood Outside (PRO-ECO) 2.0 study is a community-based research partnership with Indigenous Knowledge Keepers and Elders, Indigenous and early childhood organizations, early childhood education faculty, ELCC centers, and families, aiming to expand outdoor play in ELCC centers. This paper provides an overview of the community-based design process, guided by the 5 R’s—Respect, Relevance, Responsibility, Reciprocity, and Relationship—and the resulting study protocol for the mixed methods waitlist control cluster randomized trial.

**Methods:**

This study considered a 5 R’s research approach from its inception, beginning with the formation of a Steering Committee and over a year of relationship building before formal study activities commenced. A key early focus was collaboratively identifying project values through an iterative process. Collectively, we worked to promote equity, disrupt power dynamics, and embed Indigenous data sovereignty principles into research agreements, marking a significant departure from traditional Western research processes. The PRO-ECO program and study protocol are implemented in partnership with 10 ELCC centers delivering licensed full-day, year-round care to children aged 2.5-6 years in rural and urban areas of British Columbia, Canada. The PRO-ECO program includes 4 components to address common barriers to outdoor play in ELCC settings. Primary outcome measures include the proportion and diversity of observed nature play behavior during dedicated outdoor times at ELCC centers as measured through observational behavior mapping. Secondary outcomes include changes in educator attitudes, quality of ELCC outdoor play space, and children’s perspectives of their experiences at ELCC centers. Outcome data are collected at baseline, and 6 months and 12 months post baseline. The community’s perspectives (educators, children, and families) on the project are assessed qualitatively to understand the acceptability of the PRO-ECO program. Mixed-effect models will test the effect of the PRO-ECO program on quantitative outcomes. Qualitative data will support the interpretation of quantitative findings and provide evidence on project acceptability.

**Results:**

Participant recruitment for this study began in August 2023, and data collection was completed in March 2025. A total of 229 children, 91 staff and early childhood educators, and 40 family members were recruited to participate in this study.

**Conclusions:**

The PRO-ECO 2.0 study uses a rigorous experimental design within a community-based research project. The 5 R’s approach grounded our work in shared values, disrupting traditional academic power relations and weaving together Indigenous and Western worldviews in the context of academic research.

**Trial Registration:**

ClinicalTrials.gov NCT05626595; https://clinicaltrials.gov/study/NCT05626595

**International Registered Report Identifier (IRRID):**

DERR1-10.2196/77956

## Introduction

### Positionality

The authors of this paper include academic researchers (MB, RR, SG, DM, EF, SH, and YL) and members of the project Steering Committee (SEJ, JL, EE, EM, AL, LS, AB, EC, and AR). MB, RR, DM, EF, SH, and YL are settlers to North America, with diverse cultural backgrounds (Latin American, White European, and Asian), working within a Western academic environment at one of the largest universities in Canada. SG is Métis, Cree-French, and Scottish, working within a Western regional university that trains early childhood educators. The academic researchers’ motivation for writing this article was to describe our experiences in designing and implementing a randomized controlled trial while Indigenizing the research and working toward decolonizing the research process—identifying the power structures imposed by colonialism and working to disrupt them [1]. We seek to share our experience being led and guided by a steering committee that is predominantly composed of Indigenous members and how this profoundly shaped our research. As many researchers seek to Indigenize their research and universities enact strategic plans that emphasize decolonization and reconciliation with Indigenous people of the lands we inhabit, we hope this work can help others navigate their own journeys.

Steering Committee members, as integral partners from the beginning of the project, have important voices to include, and those who were interested have contributed to this publication and are named as authors, providing their positionality with respect to this project. Knowledge Keeper SEJ is Métis and Cree and sees the importance of time outdoors for sharing her culture and language with children. Knowledge Keeper JL is Métis and was inspired by witnessing the many gifts that being outdoors brought children. EE is a settler, early childhood educator, and researcher. EM is of settler descent and Executive Director of Early Childhood Educators of British Columbia (ECEBC), a non-Indigenous organization working with Indigenous partners. AL is of settler descent and a program manager with ECEBC. LS is Métis and, with longstanding roots in the early childhood field, has a strong connection with the land and has witnessed the many benefits of sharing this with children. AB is a family representative on the Steering Committee and a First Nations member of the Treaty 8 Territory. EC and AR are non-Indigenous family representatives on the Steering Committee. Further details on Steering Committee members are available in Multimedia Appendix 1.

### Outdoor Play in Early Learning and Child Care Settings

Outdoor play has always been a fundamental part of childhood [2], and the importance of play is enshrined in the UN Convention on the Rights of the Child [3]. Children’s participation in outdoor play, particularly in nature, nurtures well-being, helps build self-esteem and emotional intelligence, can enhance attention spans, promote feelings of autonomy and confidence, and help children connect to nature and the land and understand their role as part of the natural world [4-7]. While research reinforces the importance of outdoor play for most aspects of children’s lives [8,9], it is increasingly disappearing from their daily experiences. Children in Western nations have dwindling outdoor exposure, partly attributed to car-centric municipal design and the changing nature of neighborhoods, shifting familial priorities, and heightened technology usage [10-12]. In Canada, the alarming decline in outdoor play has led to the release of several position statements highlighting the benefits of outdoor play and urging an all-of-society approach to counteract this decline [13-15]. Included in their recommendations are suggestions to enhance outdoor play opportunities in schools and early learning and child care (ELCC) centers.

ELCC centers are a critical part of many countries’ early years landscapes, with an average of 86% of children aged 3-5 years in OECD countries enrolled in programs [16]. These settings can provide unique outdoor play experiences, supplementing children’s home and community environments. Despite the recognized importance of outdoor play, disparities persist in outdoor play implementation across ELCC programs. Many centers struggle with ensuring quality and engaging outdoor play due to various hurdles evident in their socio-ecological environments, including individual, interpersonal, organizational, policy, and societal factors, such as limited training, inadequate outdoor spaces, safety concerns, regulatory hurdles, and limited support from parents and caregivers [17-19]. Accordingly, increasing ELCC centers’ capacity to support high-quality outdoor play experiences necessitates a multifaceted approach that addresses the barriers and challenges across the socio-ecological environment of ELCC centers [17].

Previous studies that have implemented play-based interventions in ELCC centers focused on increasing physical activity and typically include free play as a supplementary component to other strategies, such as structured activities [20,21]. There is very limited evidence on effective interventions seeking to increase participation in outdoor play. To fill this gap, we conducted the Promoting Early Childhood Outside pilot study (PRO-ECO 1.0) between 2021 and 2022 in partnership with the YMCA of Greater Vancouver (YMCA) [22,23]. As part of this work, we developed and implemented the PRO-ECO program, a comprehensive outdoor play intervention to address the various barriers to outdoor play across the socio-ecological environments of ELCC centers, including individual, social, organizational, environmental, and policy factors. This program was grounded in social cognitive theory [24], and we used implementation mapping [25] to develop an implementation plan and identify behavior change techniques suited to the contexts and needs of the participating ELCC centers. We used a waitlist control cluster randomized trial design to evaluate the PRO-ECO program in 8 ELCC centers operated by the YMCA. Our experiences with the PRO-ECO 1.0 study highlighted the considerable variability across ELCC centers, even when they were part of the same organization (YMCA), and important findings related to how this heterogeneity influenced program implementation and fidelity, as well as data collection and interpretation of findings [23]. The PRO-ECO program was designed with this heterogeneity in mind, and centers were able to adapt it to their needs, while still implementing the core factors that research indicates need to be addressed to influence the provision of outdoor play in ELCC settings.

### The 5 R’s of Indigenous Research

Among the learnings from PRO-ECO 1.0 was the importance of weaving in Indigenous ways of caring for the land with Western scientific approaches. As we face the harmful effects of colonization and our role as researchers in perpetuating it, we reflect on how we conduct research to enact principles of decolonization and contribute to reconciliation and reparation efforts. As one example, existing academic discourse around outdoor play reflects a Western worldview that can also serve to perpetuate colonial ideals. Many academic writings, including those of our research team, can instrumentalize outdoor play as a means to support other outcomes (eg, physical activity, development) rather than as an end goal in its own right [10]. Relatedly, children are treated as “becomings” with interventions designed to help them reach a productive adult ideal, thereby justifying current investment, rather than “beings” and valuable members of the community in the here and now [10,26]. This is at odds with the views and traditions of the Indigenous peoples of the very lands that children are being encouraged to play outdoors upon. Rather, many Indigenous nations celebrate children as a collective responsibility of and in symbiotic interrelationship with nature, the land, and the community that surrounds them. Place is held as sacred, and in an intimate relationship with the current inhabitants and their ancestors [27].

Driven by the importance of decolonizing our research approach and of learning from and being guided by the Indigenous peoples of this land, we, as academic researchers, worked toward doing things differently. Guided by the 5 R’s of Indigenous research (5 R’s), Respect, Relevance, Responsibility, Reciprocity, and Relationship, we sought to lift Indigenous cultural values and beliefs, as reflected by the Steering Committee members and partner communities, and to disrupt and unsettle traditional academic models and power relations in our community-based research partnership. The 5 R’s served as guiding principles to help navigate this complex task. The 4R’s were introduced by Indigenous scholars Verna Kirkness and Ray Barnhardt, to foster empowerment of Indigenous people, bands, tribes and nations and include Respect for Indigenous knowledge, cultural values and traditions; conducting research that is Relevant to Indigenous perspectives and experiences; fostering Reciprocal relationships wherein the community and the researchers collaborate on every stage of the research from design through to sense-making of the emerging knowledge and mobilization of the results, providing support and resources to community members to ensure the integrity of the process; Responsibility as researchers to act ethically and ensure that every aspect of the research promotes self-determination of the community [28]. Restoule (2008, as cited by Peterson and Robinson [29]) proposed that these components can only be fully realized through nurturing and maintaining reciprocal, sustained and mutually beneficial Relationships—the fifth R. We adopted the 5 R’s to guide the entire research process, cultivating egalitarian, respectful relationships with the Steering Committee and ELCC center communities and together creating new ways of conducting research that establish greater equity. Herein, we describe our process and how it shaped the protocol for the PRO-ECO 2.0 study.

### Goal and Objectives

Our goal is to use a 5 R’s approach to design, implement, and evaluate the PRO-ECO 2.0 program and community-based randomized trial study. We were guided by the following objectives:

1. To partner with Indigenous communities, ELCC organizations, and Knowledge Keepers to adapt and implement the PRO-ECO 2.0 program in a way that is relevant to local ELCC center communities, upholds their ways of knowing, and promotes self-determination.
2. To partner with Indigenous communities, ELCC organizations, and Knowledge Keepers to co-design the randomized trial to evaluate the PRO-ECO 2.0 program.
3. To work with the participating ELCC centers to implement the randomized trial and evaluate the PRO-ECO program based on the criteria and methods identified through our partnership.

## Methods

### Overview

Our methods are reported following the recommendations outlined in the CONSIDER statement for health research involving indigenous peoples and knowledge [30], the CONSORT (Consolidated Standards of Reporting Trials) statement for cluster randomized controlled trials [31], and the SPIRIT (Standard Protocol Items: Recommendations for Interventional Trials) statement for clinical trial protocols [32]. Guidance from the Medical Research Council’s framework for developing and evaluating complex interventions [33] informed the development of the PRO-ECO program and the selection of evaluation metrics, including assessments of its contribution to real-world applications and the feasibility of the program. In this section, we outline the methodological approach, guided by the 5 R’s of Indigenous research, and then detail the waitlist control cluster randomized control trial study design.

### Ethical Considerations

We received ethics certification for this study from the University of British Columbia (UBC) and the Children’s and Women’s Health Centre of British Columbia Research Ethics Board (H20-01362). All participants in this study were required to give written informed consent. Child participants obtained written parental consent and ongoing assent during the interview process.

### Co-Designing the PRO-ECO 2.0 Study Using the 5 R’s of Indigenous Research

To ensure this project was community-guided and that our process was reflective of the 5R’s from inception, the first year of the project was spent forging relationships with and gathering input from Indigenous-led child care organizations and other partners, ELCC centers, and communities. This component of the project focused on relationship and trust building, co-designing the PRO-ECO project and research study, developing research partnership agreements with participating ELCC centers, and establishing data management and stewardship protocols, consistent with Indigenous data sovereignty principles of data governance (eg, OCAP® [Ownership, Control, Access, Possession] [34]).

### Research Governance, Prioritization, and Relationships

We convened the PRO-ECO 2.0 Steering Committee at the start of the project in early 2022. The Steering Committee is comprised of Knowledge Keepers who provide cultural knowledge and wisdom to this project and representatives from the ECEBC, Early Childhood Pedagogy Network (ECPN), Métis Nation British Columbia (MNBC), the Aboriginal Head Start Association of British Columbia (AHSABC), and the BC Aboriginal Child Care Society (BCACCS), faculty from institutions training early childhood educators (ECEs), and parents/caregivers of children attending the participating ELCC centers. Further details are available in Multimedia Appendix 1. By establishing a Steering Committee, we sought to co-create an inclusive, locally guided, sustainable, and culturally relevant supportive program for outdoor play in ELCC centers, weaving in Indigenous ways of caring for the land. Steering Committee gatherings were hosted monthly and provided opportunities to deepen relationships, engage in project planning, and establish the framework for the study. Each gathering incorporated a welcoming and closing prayer offered by our Knowledge Keepers, as well as opportunities to check in with one another, share personal stories, and receive important updates. Most gatherings occurred via web-based platforms, with 2 occurring in-person. Our in-person gatherings incorporated sharing food, smudging, and spirit plate offerings. An overview of the co-design process, highlighted through 2 years of Steering Committee connections, is outlined in Table 1.

**Table 1:** Promoting Early Childhood Outside (PRO-ECO) 2.0 Steering Committee: an overview of connections and actions completed over the study timeframe.

Timeframe	Connections	Actions
June to August 2022	First convening of Steering Committee (web-based)	Funding announcement Study design ideas Development of the Steering Committee Terms of Reference
September 2022	Connecting in Victoria, BC (in-person)	Project visioning Storytelling, connecting, and knowledge sharing
October to December 2022	Steering Committee connecting (web-based)	PRO-ECO values that guide our work Center recruitment considerations
January to May 2023	Steering Committee connecting (web-based)	Development of working subcommittees Center recruitment commencement Learning Outside Together program introduction BC Cancer shade considerations
June 2023	Connecting with participating ELCC^a^ centers in Vancouver, BC (in-person)	Connecting with ELCC centers PRO-ECO values confirmation Research agreement initiation Methodology and data considerations
July to September 2023	Steering Committee connecting (web-based)	Research agreements completion Training on data storage and sharing Data collection tools finalized ECE^b^ training program (Learning Outside Together) finalized
October to December 2023	Steering Committee connecting (web-based)	Celebrations: PRO-ECO 2.0 project officially underway! ELCC center environment modifications guidance development Presentation to the university on the unique partnership of the Steering Committee
January to April 2024	Steering Committee connecting (web-based)	Baseline data collection complete Feedback from participating ELCC centers Progress on PRO-ECO program implementation
January to April 2024	Steering Committee connecting (web-based)	Baseline data collection complete Feedback from participating ELCC centers Progress on PRO-ECO program implementation
May to August 2024	Steering Committee connecting (web-based)	Welcoming family partners Time 2 data collection updates Overview of environment modifications at group 1 ELCC centers (photos and stories)
September to December 2024	Steering Committee connecting (web-based)	Time 3 data collection update Overview of environment modifications at group 2 ELCC centers (photos and stories)
January to March 2025	Steering Committee connecting (web-based)	Celebrations: PRO-ECO 2.0 project complete! Celebrating each ELCC center and their success Early results from the PRO-ECO 2.0 study

^a^ELCC: early learning and child care.

^b^ECE: early childhood educator.

**Figure 1:**
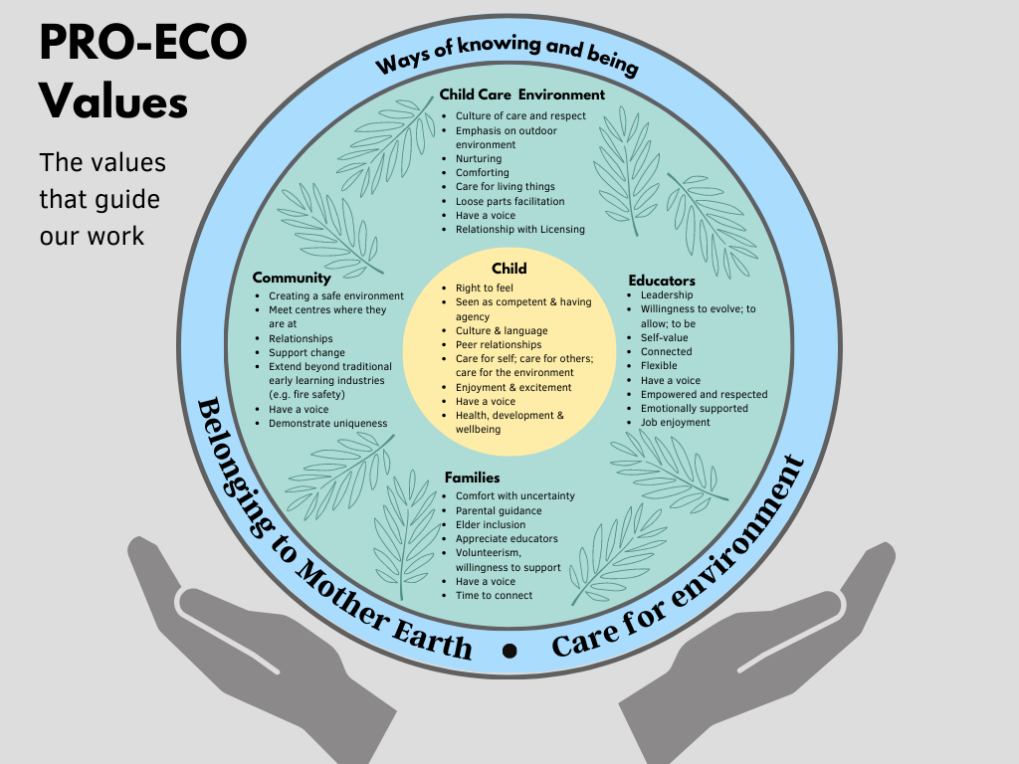
PRO-ECO 2.0 project values developed by the steering committee to guide the study and program development, implementation, and evaluation, 2022-2025. PRO-ECO: Promoting Early Childhood Outside.

A foundational component of our early work together was identifying project values. The project values identified by the Steering Committee emerged through an iterative process that spanned a year and can be seen in Figure 1. Children are centered in this work, supported by their communities, families, ECEs, and the ELCC center’s social and physical environment. Within each category, the Steering Committee identified the elements they wished to be supported and emphasized as part of the PRO-ECO 2.0 study. Surrounding all values is the connection and relationship with the Land, as well as care for the environment, grounded in local ways of knowing and being. These values guided all aspects of the project, including research questions, primary outcomes, data collection methods, inclusion criteria, data analysis, and interpretation.

### Research Agreements and Indigenous Data Sovereignty

We entered into formal research agreements with each participating ELCC center in 2023. These agreements were developed and signed on our behalf by the Innovation University of British Columbia’s (iUBC). We worked with iUBC and UBC’s Indigenous Research Support Initiative to establish greater equity and disrupt power dynamics, incorporating the principles of Indigenous data sovereignty into the research agreements. This represented a considerable departure from the template wording of these research agreements. We developed 2 versions: one for Indigenous communities and one for non-Indigenous communities. Both versions specified that ownership of the data was retained by the ELCC communities, with stewardship by the UBC. The sole difference between the agreements related to ensuring the Indigenous partners retained their “moral rights,” that is, the rights of the Indigenous partners to be associated with the Indigenous knowledge, and ensure the accuracy and cultural sensitivity of interpreting the Indigenous knowledge. In practice, this involved requiring prior written consent for any requests to extend data use rights, or to modify or adapt Indigenous knowledge, as well as attributing ownership of the Indigenous knowledge to the Indigenous partner in all publications. The draft research agreements were shared with communities as a starting point for conversations to incorporate their input in shaping the terms included within, such that it was possible to end up with different agreements for each community. We held conversations with the leaders identified by each community as responsible for signing the agreements and adapted them according to their needs.

Importantly, the research agreements prioritized data sovereignty, guided by data sharing and ownership principles in line with OCAP®. This supported participating ELCC centers and organizations to have control over their data collection process, ownership of their data, and the interpretation and use of their data. All collected data were shared and stored on a secure software platform administered through UBC, which facilitated ownership and access of data by each ELCC center, and stewardship of data by the research team. This process proved resource-intensive as it required additional training with each ELCC center on the use of the data sharing software, as well as ethical considerations when working with the data.

### PRO-ECO Study: Traditional Scientific Model Versus 5 R’s of Indigenous Research Approach

The PRO-ECO 1.0 study conducted in 2021 and 2022 followed a traditional scientific model in which we used a waitlist control cluster randomized trial design to implement and evaluate the PRO-ECO program. During this study, we worked with the YMCA management team to plan and conduct the study and interpret the results. While we sought input from the management team in key decisions, such as selection of ELCC centers, and of the primary outcome, the academic team, guided by a scientific advisory committee, designed the study and selected the methods and measures. The YMCA management team selected the 8 ELCC centers to participate in the study, with centers informed that they were being included in the research, rather than volunteering to participate. Further details on our approach and methods have been published elsewhere [22].

This top-down approach is in contrast to the 5 R’s research approach that was used to plan and implement the current PRO-ECO 2.0 study. Although we used a waitlist control cluster randomized trial design to evaluate the PRO-ECO 2.0 study, as was done in the PRO-ECO 1.0 study, important differences emerged as a result of the 5 R’s research approach, reflecting our weaving in of Indigenous knowledge and ways of knowing. These differences included the design and implementation of the PRO-ECO program, selection of the primary outcome and methodology for the study design, and data ownership and stewardship agreements. Multimedia Appendix 2 provides a complete list of how the PRO-ECO 2.0 study differed in comparison to the PRO-ECO 1.0 study.

### Implementation of the PRO-ECO Program

The PRO-ECO program was designed to address the barriers to outdoor play in ELCC contexts identified in previous research with ECEs [17,18]. Many challenges relate to ECEs’ attitudes toward outdoor play and sense of self-efficacy in facilitating outdoor play and managing the risks inherent in it, such as children’s risk-taking [35]. They have further emphasized the importance of having a common philosophy and approach with their colleagues and the managers of the ELCC with regard to pedagogical decisions and allowances for risk in play. The ECEs also highlighted that if parents and caregivers of the children understood why outdoor play was important for their child, then they were less likely to challenge outdoor play during inclement weather, perceive outdoor play as a waste of time better spent on academics, or be excessively concerned with the perceived risks of the outdoor environment [17].

The PRO-ECO program was first developed and implemented in the PRO-ECO 1.0 study and included 4 primary intervention components: ELCC center outdoor play policy, ECE training, ELCC center outdoor space modification, and parent engagement. To develop the PRO-ECO program, we used intervention mapping [36] and behavior change techniques [37] guided by social cognitive theory (SCT) [24] to address the identified challenges and build the skills necessary to support outdoor play in ELCC settings [38]. Each of these 4 components can be modified according to the needs and context of individual ELCC centers. Through the collection of baseline data, the program is further refined to provide center-specific adjustments, such as specific materials in the built environment design modification or targeted follow-up training and mentorship (Table 2). The PRO-ECO 1.0 study revealed important findings related to the implementation and evaluation of the PRO-ECO program [23]. These learnings included ensuring the sustainability and scalability of the program for lasting behavioral and environmental change, supported through continued training, mentorship, and landscape maintenance. In addition, building stronger relationships with educators, families, and children further supports the long-term integration of outdoor play in ELCC settings. Participating ELCC centers in the PRO-ECO 1.0 study were also constrained in the implementation of the PRO-ECO program at times due to staffing levels and resources.

**Table 2:** Promoting Early Childhood Outside (PRO-ECO) program components and implementation in the PRO-ECO 2.0 study.

PRO-ECO program component		PRO-ECO 2.0 implementation
**Outdoor play values, policy, and practice**		
	ELCC^a^ center administrators and staff identify the organization’s core values related to outdoor play, including why they value it, and how they want to support it. The values form the basis for a review of existing policies, procedures, and practices to determine whether they are aligned or require modification. Values are shared with the ELCC community, and the modified policies, procedures, and practices are implemented within the ELCC center.	Each ELCC center’s administrators and staff worked together on this activity. It was integrated into the professional learning and mentorship avenues, and also supported by a supplemental session with research team members.
**ECE^b^ Professional Learning and Mentorship**		
	Training and mentorship to build knowledge and self-efficacy around outdoor play. Ongoing mentorship to address emerging issues and build on learning.	The Learning Outside Together, Incorporating traditional wisdom and promising practices to futureproof child care (LOT) program is an already established 10-12-week program with asynchronous online modules that are paired with synchronous weekly meetings where ECEsb meet with a peer mentor [39]. LOT incorporates local, traditional Indigenous wisdom of Land as Teacher and practices related to outdoor learning in ELCC settings. A mentorship network, which includes liaisons from all participating ELCC centers, meets via web once per month to discuss details of the project, troubleshoot emerging issues, and share ideas. Indigenous centers are paired with Indigenous mentors to support cultural safety. ELCC centers participating in the PRO-ECO 2.0 study were invited to participate as their own cohort as part of the larger LOT program (which operates outside of the PRO-ECO 2.0 study).
**Outdoor Space Modification**		
	ELCC centers modify their outdoor space to increase affordances for play. Modifications include adding loose parts, plants, and other natural materials.	Environmental modifications were designed and implemented by each of the ELCC centers, with support from the research team and the Steering Committee. Each participating ELCC center was allocated the same funding to complete environmental modifications and retained considerable discretion in how to allocate these resources. Each center was provided with CAD $8800 (US $6471) for general expenses and CAD $1600 (US $1176) for shade-related components. Liaisons were given a workshop on the Seven Cs design guidelines [40] and provided with a “look-book” with ideas for outdoor space and shade modifications. The research team worked with each center to help support the development and execution of their plans.
**Family Engagement**		
	Each center plans a family engagement event in the format that most suits their families. The intention is to increase parents’ and caregivers’ knowledge of the importance of outdoor play, and encourage family involvement in implementing the outdoor space modification.	Materials and engagement activities led by ELCC centers with support, as needed, from Outside Play Lab. Examples of family engagement activities include: seed planting activity, working in garden boxes, planting seedlings, and creating beanpole teepees, sharing food with families and talking about the project, looking at info graphics and maps with data from interviews with the children.

^a^ELCC: early learning and child care.

^b^ECE: early childhood educator.

These learnings, alongside a 5 R’s research approach, were considered in the implementation of the PRO-ECO program as part of the PRO-ECO 2.0 study, as outlined in Table 2. For example, engagement events were mobilized in a format that best suited the families at each center to increase engagement opportunities that were often challenging to realize in the PRO-ECO 1.0 study. In addition, ECE training was implemented through a mentorship network over a period of 10-12 weeks to support more long-term learning and sustainable training opportunities. ELCC centers participating in the PRO-ECO 2.0 study also received financial resources to help defray the costs of participation. Each site received CAD $15,000 for staff costs (US $11,029), CSD $8800 (US $11,029) to pay for supplies and modifications to the general outdoor environment, and CSD $1600 (US $1176) from BC Cancer to cover the costs for shade-related materials. The ELCC centers were informed about the funding for staff costs after enrollment, when extra resources became available from the funding agency.

### Study Design

The PRO-ECO 2.0 study is designed as a mixed methods waitlist control cluster randomized trial. This mixed methods approach included quantitative and qualitative data collection to evaluate the effects of the PRO-ECO project, and qualitative data gathering to plan project implementation, understand children’s ECEs’ and families’ experiences with the project, and gather their perspectives on its effects.

### Inclusion Criteria and Recruitment

The PRO-ECO project was designed for ELCC centers that provide licensed full-time group child care to children aged 2.5 to 6 years. The Steering Committee decided to open the study to ELCC centers in BC that opted into the Affordable Child Care Benefit, which is a provincial government subsidy to support eligible families with the cost of child care [41], to help ensure inclusivity of lower-income families. Additionally, while we wanted to be as inclusive as possible of remote rural locations, practicalities necessitated that ELCC centers had reasonable access to a plant nursery or hardware store to be able to access material for the outdoor space modification. The definition of what was reasonable was left up to the ELCC centers to determine (eg, within a 2-hour drive).

Recruitment notices were delivered through our Steering Committee partner organizations (in newsletters or through email blasts), posted on social media, and on the Outside Play Lab website (OutsidePlay.org). While recruitment was not explicitly targeted toward Indigenous-led ELCC centers, by distributing through our Steering Committee and partner organizations, we received interest from more Indigenous-led centers than might have been the case if the notices were distributed by another, less known or trusted organization. Interested ELCC centers contacted the Outside Play Lab, and an informal meeting was planned to explain the parameters of the partnership. A secondary zoom interview, with multiple PRO-ECO steering committee and center/band representatives, to discuss readiness for partnership, participation, and particularly access to resources to support the necessary time to the project (ELCC centers could rely on volunteers, managers, Elders, or others within their community to help them meet these time commitments). Discussion questions were provided before the meeting, and ELCCs had the opportunity to respond with their items before and during the Zoom session. The meeting also offered an opportunity to introduce members of the PRO-ECO team and their roles, while touching on how management, including staff teams, boards or organizations, and community bands or municipal governments, could modify policies, practices, and outdoor play environments.

A total of 28 ELCC centers expressed their interest in participation, and of these, 12 ELCC centers were selected for inclusion. While our desired sample size (see sample size calculation below) required the recruitment of 10 ELCC centers, we selected 12 ELCC centers to account for potential center withdrawal. Indeed, 2 centers decided not to continue participation prior to baseline data collection, citing staffing challenges. Following site selection, each ELCC appointed an ELCC-PRO-ECO project liaison (“liaisons”) to connect the research team, ELCC staff, and families. ECE’s and staff at participating ELCC centers during the course of the study were recruited to participate in this study between August 2023 and October 2024.

The PRO-ECO 2.0 study included ELCC centers in 10 different communities across the South Cariboo, East Kootenay, Northern, Southern Interior, Greater Vancouver, Thompson Okanagan, and Vancouver Island regions of British Columbia, Canada. Among the 10 participating ELCC centers, 7 are located in rural locations and 3 in urban locations. In addition, 5 participating ELCC centers are operated on reserve by Indigenous-led organizations.

Children attending a participating ELCC center during the course of the study and their family members were recruited to participate in this study between September 2023 and October 2024. Recruitment of children and families occurred through each ELCC center, posting flyers and distributing letters and emails that outlined details of the PRO-ECO project and sought participation. Center staff also initiated face-to-face conversations with families during drop-off and pick-up times. Over the course of the study, participating children left or graduated from their ELCC center, and newly enrolled children were recruited as the study progressed. Parental written consent was received from participating children. All children attending the ELCC programs were exposed to the PRO-ECO program, either as group 1 or group 2, regardless of consent status.

### Randomization

Individual ELCC centers served as the unit of randomization in the waitlist control cluster randomized trial design. ELCC centers were randomized to either group 1 or group 2 prior to baseline data collection. Five group 1 centers implemented the PRO-ECO program immediately following baseline data collection, whereas 5 group 2 centers, acting as waitlist control sites, implemented the PRO-ECO program following time 2 data collection. Randomization was conducted by Steering Committee members who randomly drew 5 separate slips of paper with ELCC center names to represent group 1. The research trial coordinator was not blinded to the randomization, and ELCC centers were aware of their group during baseline data collection because planning was already underway with group 1 ELCC centers for launching the implementation of the PRO-ECO project. Data on outcome measures were collected at 3 time points: baseline (time 1), 6-month follow-up (time 2), and 12-month follow-up (time 3). Outcome data aimed to assess the short-term and longer-term outcomes within group 1 and the short-term outcomes within group 2. Data collection occurred during the fall and spring seasons to compare similar weather patterns at each data collection time point.

### Data Collection and Measures

#### Primary Outcomes

Led by the values they identified as guiding the study (Figure 1), the Steering Committee sought to emphasize children’s relationship with the land and care for the environment. Thus, study outcomes were prioritized to understand how the PRO-ECO project influenced children’s engagement with the land. As a result, the primary outcome chosen for the PRO-ECO 2.0 study was the occurrence of outdoor nature play behavior at ELCC centers. While the primary study outcome differs from the PRO-ECO 1.0 study, where the occurrence of play versus nonplay was assessed, children’s outdoor play behavior was still coded using the expanded version of the Tool for Observing Play Outdoors (TOPO) [42]. The TOPO is designed to measure 8 play types (physical play, exploratory play, imaginative play, play with rules, bio play, expressive play, restorative play, and digital play) and one nonplay type, along with corresponding subtypes. For this study, we coded nature play behavior through the TOPO bio play behavior type, which includes subtypes for play (observing; discussing; or interacting with plants, wildlife, or natural materials, as well as playing in a way that demonstrates care or stewardship of the environment or appreciation of nature). To further refine observed nature play behavior, children’s environmental interactions (loose parts, fixed parts, natural elements, manufactured elements) were also recorded and coded.

The TOPO was implemented through a child-based, observational behavior mapping (OBM) protocol. The purpose of OBM is to understand how an environment supports movement and play behavior by mapping, recording, organizing, and analyzing geographically located data (37). Base maps were developed for each center and uploaded onto digital tablets to facilitate geo-located coding of play behavior. Due to the rural locations and varied geographic distances of most participating ELCC centers, the research team did not code play behavior in situ as was done in the PRO-ECO 1.0 study. Further, in situ coding requires significantly more time, and obtaining reliability in coding requires extensive training, making it unfeasible to have ELCC staff code the play behavior directly on-site. As a result, the liaisons at each center were trained to observe and video record children while outside during scheduled outdoor time. Children were recorded according to a systematic protocol where the liaisons at each ELCC center had a list of consented children. They then captured a 2-minute video of the child’s outdoor play and verbally dictated what they noticed about the play during the video observation. We aimed to collect a total of 64 two-minute videos for each ELCC center at each data collection time point (time 1, time 2, and time 3). The observational data were collected at the child level, where individual children were the unit of observation and observations aimed to capture all consenting children. This differs from the PRO-ECO 1.0 study, which collected observational data at the center level to study children’s outdoor play in connection to their ELCC. Following the collection of videos at each time point, research staff split each 2-minute video into 15-second clips (512 video clips at each center for each time point). Trained and reliable coders coded each 15-second clip to assess the TOPO play types and subtypes. The reliability of the OBM method is defined by the degree of interrater reliability and interrater agreement, which was assessed using weighted κ and intraclass correlation coefficients [43,44]. κ Values of ≥0.70 are commonly accepted as adequate for scientific research [43]. A κ value of 0.82 was achieved between coders in relation to the primary outcome variable (TOPO) prior to beginning video coding.

Additional variables were collected through the OBM protocol in this study, including risk-taking behavior, activity intensity, peer interaction, adult interaction, and play communication. Further details on the OBM protocol and process are available in the PRO-ECO 1.0 study protocol by Ramsden et al [22].

#### Additional Outcome Measures

##### ECE Attitudes Toward Outdoor Risky Play

To understand how ECEs’ attitudes toward outdoor risky play may shift over the course of the study, we measured educator perspectives using the Teacher Tolerance of Risk in Play Scale (T-TRiPS) [45]. The T-TRiPS is a 25-item instrument and has been validated for use as a measure of intervention effects aimed at increasing children’s access to risky play (a fundamental component of outdoor play). Questions include items that range from those that most ECEs should have little trouble endorsing (eg, “Do you let children in your center play chase with one another”) to those that they might find more challenging (eg, “Do you let children in your center use adult tools [eg, hammer and nails, knife, scissors] unsupervised?”), with the response options being “yes” or “no.” ECE’s at participating ELCC centers completed the T-TRiPS at time 1 and time 3.

##### Quality of ELCC Outdoor Spaces

Modifications to the outdoor environment were guided by the Seven Cs design guidelines [40]. A simplified version of the Seven C’s was developed by the research team, as seen in Table 3, in order to facilitate its completion by ELCC staff. At time 1 and time 3, center staff were encouraged to complete the Seven Cs together, centering the perspective of the children at their center. In addition to acting as a baseline measure, the time 1 measurement provided guidance to the centers regarding elements of the outdoor environment that are working well and those that might require improvements. Subsequent measurements indicated how the modifications impacted the quality of the ELCC outdoor spaces.

**Table 3:** Seven Cs characteristics and ratings adapted from Herrington and Lesmeister [40] and Brussoni et al [46].

Characteristic	Rating (5=strongly agree, this is well done in my center; 4=somewhat agree; 3=neutral; 2=somewhat disagree; 1=strongly disagree, this is lacking in my center)
Character	The entire space feels welcoming for the children and ECE (eg, soft materials, dappled light, some color)
Context	The property or area beyond the play space fence contributes positively to the atmosphere of the play space
Connectivity	The children can move seamlessly from one area of the yard to another (eg, defined pathways for tricycles, and pathways for foot traffic only, such as stepping stones or tree cookie paths)
Clarity	Different zones of play are clearly identifiable at the child’s height (eg, messy zones, sand play, water play, tricycle space)
Chance	Throughout the play space, there are natural and loose materials to create and build with, manipulate, and move with
Change	There are ample amounts of natural features, such as trees, shrubs, and groundcovers, in the play space (or directly adjacent to the play space) that change with the seasons
Challenge	The play space encourages different levels of physical and cognitive risk-taking (eg, height, speed, using tools, hiding/getting lost, rough-and-tumble)?

##### Children’s Perspectives

As evident in the PRO-ECO values seen in Figure 1, the Steering Committee centers the child and their wellbeing, surrounded by the caregivers and community that support them. Steering Committee members emphasized the importance of hearing directly from children about their perspectives. They also stressed the importance of strength-based measures, rather than deficit-based measures. This was in contrast to our approach in the PRO-ECO 1.0 study, where we had relied on questionnaires completed by ECE staff about the children, which examined difficulties, such as conduct problems, aggression, and depressed affect.

For this study, we adapted the Early Childhood Education and Care Well-being Monitor, a structured interview designed for ECE staff to gather the input of children aged 4 to 6 years regarding their well-being in the ELCC center [47,48]. It includes questions about the children’s daily experiences at the center (eg, “Do you think it’s fun here?”), their activities (eg, “Tell me about some of your favorite things to do here.”), relationships with other children (“eg, Do you have some good friends here?”) and the ECEs (eg, “Do you want adults to play with you more?”), the physical environment (eg, “What is your favorite place outdoors here?”), and the extent to which their views are listened to (eg, “Do the adults decide what you are going to do when you are outdoors here?”). For this study, we eliminated questions related to the indoor physical environment and adapted the remaining questions to suit our study’s context, for a total of 23-25 questions, depending on the time point (pre- or postprogram). These questions guided semistructured interviews with each child, and we referred to the mosaic approach for listening to young children to further facilitate children’s participation [49]. For example, children were shown images of 3 faces, a green smiley face, a yellow neutral face, and a blue frowny face, which they could point to for each question to support their responses.

Child interviews were conducted for each individual child on-site by the ECEs at the participating ELCC center. Due to the time and resources required to conduct interviews with children, we collected these data at time 1 (preprogram) and time 3 (postprogram) only. ECEs identified a child from the consented study participant list and found a quiet place, preferably outside, to conduct the interview. Using a video camera, ECEs introduced the interview activity to the child and gained the child’s assent to ask them questions. A child could remove assent at any point by declining to continue. Following the interview, ECEs noted down notable features of the interview, such as: if the child moved around or was distracted; if other children joined in the interview; if the child made body language movements that the audio would not capture; or to reflect on impressions and note down some immediate thoughts. For children who required additional support or nonverbal cues, emotion icons and images of different features in the child’s ELCC center’s outdoor space were provided. In addition, some ECEs found that walking around the outdoor space with the child during the interview could facilitate responses. ECE’s conducting interviews were trained to stop the interview if the child removed assent and not to force an interview if a child did not wish to participate.

##### ELCC Center Staff Perspectives

To gather staff perspectives on the PRO-ECO program, we conducted listening circles with each ELCC center at baseline (time 1) and after the center implemented the PRO-ECO program (time 2 for group 1 and time 3 for group 2). At baseline, questions that guided the listening circles included their perceptions of outdoor play and the outdoor space in their ELCC center, the current practice and pedagogy of outdoor play, their role in supporting outdoor play, and recommendations for implementing the PRO-ECO program. At the postimplementation listening circle, we queried the changes they noticed, what they liked and did not like, how the program changed their practice, and any notable observations relevant to the effect of PRO-ECO on the children and family engagement. The listening circles were conducted in ways that best fit the ELCC center needs, such as scheduling them during already existing staff meetings. Staff time was covered by the funding provided to each center.

##### Family Perspectives

To gather family perspectives on outdoor play changes they may have noticed after the implementation of the PRO-ECO program at their child’s center, we provided a brief survey and conducted short one-on-one interviews in-person and over Zoom. We sought their reflections on any changes they may have noticed in their children, the ECEs’ approach to outdoor play, and the outdoor space. Each survey participant was offered a CAD $10 (US $7) gift card, and interview participants received a CAD $50 (US $37) honorarium.

### Study Covariates

We collected data on the following covariates that have been found to influence children’s outdoor play: children’s demographics, including sex, age, disability status, first language spoken at home, family composition, average household income, and highest level of education completed by a household member. We collected information related to the child’s length of time at the ELCC center and their experiences with any other forms of formal care. This information was gathered through questionnaires completed by families at the time of the child’s enrollment in the study.

Weather conditions (eg, sunny, cloudy, heavy rain, snow) were recorded by the liaisons at the beginning and end of their play video recording sessions. Temperature data were recorded by the research team using weather network databases for the weather station closest to the ELCC center.

The following information related to recruitment, retention, and attendance was collected throughout the study:

- Number of eligible children that were approached to participate in the study.
- Number of children who consented to participate, did not consent to participate, and did not respond.
- Number of children who withdrew from the study.
- Number of children who enrolled in the ELCC center after initiation of the PRO-ECO program.
- Number of children who left the ELCC after initiation of the program.
- Individual and center-wide attendance rates.

### Data Analysis

We aimed to collect 512 fifteen-second video clips at each center for each time point, for a total of 5120 video clips per time point (total N=15,360). This power calculation was based on the statistically significant effect size we observed in the PRO-ECO 1.0 study related to the short-term impact of the program on improving play occurrence (time 1 vs time 2 for group 1 and time 2 vs time 3 for group 2) [23]. The key coefficient of interest is the intervention by group interaction term, which corresponds with a *R*^2^ increase of 0.00295 (ie, variance explained by the special effect), equaling to an effect size of 0.00308, when this interaction term is added to the multivariate regression model. We also expect to have 5 control variables (ie, intervention, group, weather conditions, temperature, and child gender) in the multivariate regression models, which altogether correspond with a residual variance of 0.958. This supports a power calculation of over 99% (0.997) to detect the program effect that we observed in the PRO-ECO 1.0 study. The power calculation was conducted using the G*Power software [50].

To analyze the effect of the PRO-ECO program within the PRO-ECO 2.0 study, we intend to evaluate the change in nature play behavior between pre- and postprogram. The proportion of nature play occurrences compared with nonnature play occurrences across ELCC centers at baseline will be summarized by treatment group using frequencies and percentages. This approach mirrors our analysis in the PRO-ECO 1.0 pilot study [22,23], which demonstrated the effectiveness and significance of measuring children’s outdoor play occurrences through a comparison of play and nonplay behaviors pre- and postprogram implementation. However, in contrast to the PRO-ECO 1.0 study, this study focuses on the occurrence of nature play as the primary outcome variable, and not play participation in general.

Baseline demographic and ELCC characteristics will be summarized by group using means and SDs, medians and IQRs for continuous variables, and frequencies and percentages for categorical characteristics. Bivariable relationships between children’s demographic characteristics and outdoor nature play occurrence, both overall and by group, will be explored to identify potential confounding factors at the individual level, considering randomization occurs at the cluster level. Mixed effects models will be used to assess differences in quantitative outcome measures between groups, as well as within-group comparisons of pre- and postprogram implementation measures.

We will analyze child interviews using qualitative content analysis methods to systematically identify patterns and themes that reflect children’s perspectives and experiences on outdoor play and well-being [51]. The method’s adaptability to diverse data types and research contexts, combined with its structured and systematic analytical process, makes it particularly viable for exploring the wide-ranging and expressive nature of children’s responses. For ECE and family listening circles and interviews, we will use thematic analysis, which involves identifying and analyzing key themes within the data [52]. We will follow a reflexive thematic analysis to facilitate insights regarding ECEs’ and families’ perspectives on various interconnected topics, such as the importance of outdoor play, power dynamics, cultural norms on outdoor play, and the acceptability of the PRO-ECO program.

In alignment with the PRO-ECO project values and the partner research agreements, we will work with participating ELCC centers and the Steering Committee to share ongoing results from this study. Steering Committee members and liaisons will be invited to join a working group to work with the Outside Play Lab team on guiding and interpreting the analyses. We will gather ongoing iterative input from the broader Steering Committee and ELCC center teams to help interpret the findings, guide subsequent analyses, and develop recommendations. This form of member checking will seek to ensure all analyses and results align with the desired outputs of our project partners and appropriately convey the findings.

### Knowledge Mobilization: Sharing the PRO-ECO Story

As with other aspects of the PRO-ECO study, planning for knowledge mobilization follows a 5 R’s approach and will be codeveloped with the Steering Committee and the ELCC centers, who will identify primary audiences for knowledge materials, the materials that are most suited to those audiences, and the methods for reaching them.

Steering Committee members, and where appropriate liaisons, will be supported in participating as co-presenters and coauthors for academic presentations and publications in peer-reviewed journals. We recognize that the tensions between Western academic and Indigenous worldviews are particularly notable when conducting knowledge mobilization within these traditional academic approaches. For example, the process of writing this academic publication represented a microcosm of our experiences with the project and illuminated the many pressure points when bringing together Indigenous and Western academic worldviews. Academic publication comes from the Western scientific tradition. By its very nature, it relies on written accounting (rather than oral storytelling, for example), and brings with it many assumptions and expectations on content and language use. It also requires that only authors be listed if they meet strict criteria for authorship and are listed in an order determined by their contributions [53]. Working within these norms and boundaries, we designed a process to make authorship inclusion as low-barrier as possible for our steering committee members. To facilitate their input and authorship for this publication, we shared a manuscript draft and presented it to our Steering Committee. We followed up with each member to see how we could best support their involvement if they were interested in being included as coauthors, such as planning individual conversations to describe the work and gather their input.

We recognize that knowledge mobilization through academic publications represents only one form of storytelling and reaches a scholarly audience primarily. Many diverse forms of storytelling must be integral to our knowledge mobilization efforts to reflect the different worldviews and needs of the Steering Committee and partner communities. This could include methods emphasizing oral traditions, such as videos and presentations, as well as newsletter posts, infographics, or other methods of sharing the story of the study in the places where the people we want to reach access information. We will also work with ELCC centers to develop a knowledge mobilization plan that is suited to their community, for example, family engagement events, infographics, family newsletter posts, or similar materials.

## Results

**Figure 2:**
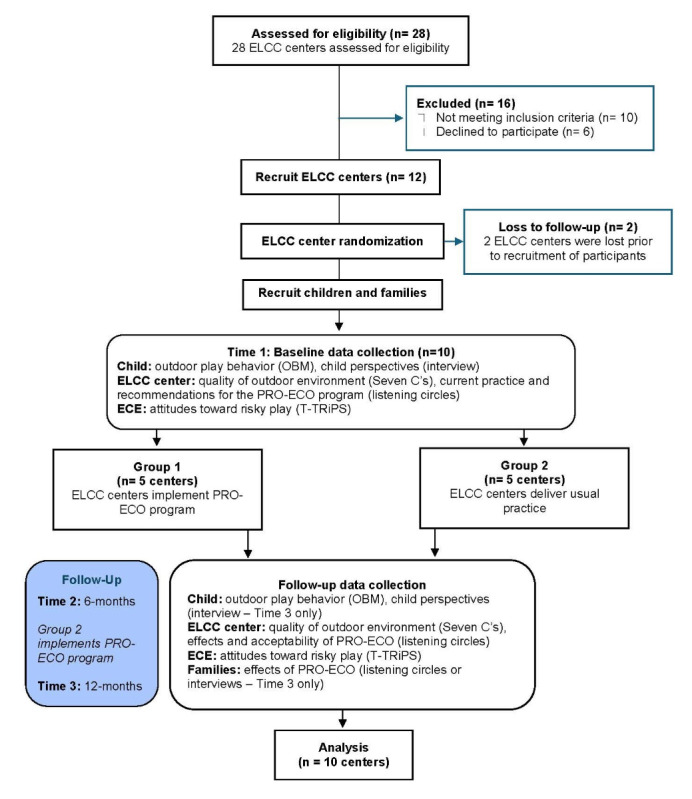
Promoting Early Childhood Outside 2.0 study: Wait-list control cluster randomized trial flow diagram. ELCC: early learning and child care; PRO-ECO: Promoting Early Childhood Outside; T-TRiPS: Teacher Tolerance of Risk in Play Scale.

This study was developed to cocreate, administer, and evaluate the PRO-ECO program following the implementation of the PRO-ECO pilot study in 2021. Funding to conduct this study was confirmed in March 2022. Ethics approval was received in September 2022. Recruitment of ELCC centers occurred from January 2023 to June 2023, followed by participant recruitment beginning in August 2023. Baseline data were collected between October 2023 and December 2023, and the group 1 ELCC centers received the PRO-ECO program from December 2023 to April 2024. Group 2 ELCC centers received the PRO-ECO between May and September 2024. Data collection was completed in March 2025. A total of 229 children were recruited to participate in the study, with 193 children participating in observational play videos for coding and 162 children participating in one-on-one interviews. Additionally, 91 staff and educators consented to participate in the study, with 59 participating in focus groups and 84 completing the T-TRiPS survey. In follow-up to consenting for their child to participate, 41 family members completed an interview or family survey to add their perspectives to the study. The PRO-ECO 2.0 waitlist control cluster randomized trial flow diagram is provided in Figure 2.

## Discussion

### Principal Findings

In this project, we use a 5 R’s approach to lift Indigenous cultural values and beliefs to disrupt traditional academic power relations, to partner together to increase children’s daily opportunities for outdoor play in their ELCC centers. Our work together is grounded in relationships and shared values that transcend individual worldviews, with a common goal to benefit the lives of children and the ELCC community that surrounds them.

There are several places where the tensions between the Western academic approaches and Indigenization and decolonization of research can seem irreconcilable, such as in choosing to use a randomized trial design for this study. Western academic traditions consider it a “gold-standard design” for evaluating the effectiveness of intervention programs, and therefore, it can be a persuasive tool for supporting change [54]. It stems from a positivist worldview with the assumption that environments and variables can be controlled and reliably measured to determine whether an intervention caused a particular effect [55]. There is a rich literature on the limitations and challenges involved in conducting randomized trials in community-based research, with some indicating that these approaches are ultimately incompatible because of divergent values or goals [56]. The importance of a long planning phase has been highlighted along with careful consideration of the evidence that can meaningfully indicate the effectiveness of the program or intervention [56].

In this project, our relationship building and deliberate process to identify project values have been a critical ingredient in working to realize a 5 R’s approach for this study. The explicit values (Figure 1) illuminated a path of decisions at key junction points, such as what was considered meaningful evidence, and from whose perspective. This approach placed children at the center and included measures that helped us include children’s voices and ensure data collection methods emphasized a strength-based perspective.

### Strengths and Challenges With the 5 R’s Research Approach

A significant strength of PRO-ECO 2.0 study is the creation of a Steering Committee to guide this work. Their steady guidance and encouragement helped us work toward realizing a 5 R’s approach for this work. We anticipate that the results of this study will provide important evidence to help inform curriculum, policies, outdoor environments, and professional development training that support outdoor play opportunities among children in ELCC centers. This study will also provide insightful alignment with international research on outdoor play in ELCC centers [57,58].

An additional strength relates to the learnings from PRO-ECO 1.0 that we were able to incorporate into this study to address some of the challenges and limitations we had encountered. We sought to include a diverse sample of ELCC centers, including demographic and geographic diversity, and this presented new challenges to the study. The vast geographic locations of ELCC centers required a different approach to data collection and data sharing than reflected in the PRO-ECO 1.0 study, including ELCC center staff collecting data themselves. These opportunities strengthened the project in many ways by enhancing project involvement for the ECEs at all stages of the study. However, it also contributed to limitations with the extent of data that could be collected in terms of quantity, approaches, and tools. ELCC communities may not have understood the amount of work involved, and this meant that in our commitment to egalitarian involvement and empowering their participation, we had to increase the anticipated resources to support them. In addition, the use of video recordings captured by ELCC center staff and later coded off-site by the research team posed challenges in accurately interpreting children’s actions and movements, given the limited sound and contextual information inherent to second-hand observation [59]. In situ coding, as implemented in the PRO-ECO 1.0 study, supported richer contextual understanding and immediate interpretation for the research team working with on data coding and analysis. However, off-site coding was necessary in this study due to the remote locations in which data were collected. Although the data coding presented more challenges in this study, potential observer bias may have been reduced by having ELCC center staff already integrated into the children’s daily environments, who conduct all observations [60].

The innovative nature of creating distinct research agreements with each ELCC community, rather than adopting a single overarching agreement typical of conventional research projects, offered substantial strengths, including prioritizing community agency and reciprocal data ownership and sharing relationships. However, it also introduced challenges within the research agreement development, including the data collection and data sharing process. Traditional Western academic institutions often prioritize data ownership within the university, supported by institutional policies and software designed to ensure ethical and secure data management, and commonly promote open-access frameworks. This study diverged from those norms by asserting that data should remain within the community rather than be institutionally owned or publicly shared. Compounding these challenges was the limited guidance in existing literature on creating research agreements that depart from conventional models. To ensure inclusivity and respect for all stakeholders, individualized data agreements were codeveloped with each ELCC center and their community, the Steering Committee, the research team, iUBC, and the Indigenous Research Support Initiative. Although time- and resource-intensive, this collaborative process was essential to ensure all perspectives were represented in the final agreements.

To further support community-based data ownership, ELCC centers received training on ethical data management and the use of centralized software systems for securely collecting, storing, and sharing sensitive child-related data. Variation in the resources available across ELCC centers to manage data ownership, storage, and sharing was evident. Some centers had sufficient infrastructure, such as reliable Wi-Fi and appropriate hardware, which facilitated smooth data collection and transfer. In contrast, ELCC centers in remote locations often faced inconsistent connectivity and limited access to necessary equipment, hindering data sharing with the research team. In some cases, team members had to travel to these sites in-person to retrieve stored data directly from hardware devices. Staffing capacity also influenced these processes; ELCC centers with more personnel, or with staff experienced in using digital tools, were better equipped to meet the project’s data management requirements.

The implementation of the PRO-ECO program also faced challenges, particularly in adhering to the designated implementation timeline. Sustaining engagement with the ECE training and introducing or maintaining built environment modifications were at times limited by staffing constraints, seasonal changes, and difficulties obtaining materials in the remote locations of participating ELCC centers. Although the PRO-ECO program was scheduled to be implemented between December 2023 and April 2024 for group 1 ELCC centers and May 2024 and September 2024 for group 2 ELCC centers, actual implementation extended beyond these periods in some ELCC centers. These challenges were consistent with those observed during the PRO-ECO 1.0 study [22,23].

### Conclusions

The PRO-ECO study provides an example of the 5 R’s in practice, in the context of a rigorously-designed evaluation of a program to support outdoor play in ELCC settings. Including diverse ELCC centers in urban, rural, and remote areas will unearth new learnings on how to support their community and practice in the context of outdoor play. In designing and conducting this mixed methods waitlist control cluster randomized trial, we sought to ensure that we worked in ethical and responsible ways to disrupt traditional power structures and promote self-determination of the participating communities, balancing the need to produce what Western scholars consider rigorous evidence, while also prioritizing in Indigenous community perspectives, centering the voices of the ELCC community and gathering rich contextual data to help understand their daily realities. In sharing our story of building and conducting this study, we hope that our experiences can provide scholars and communities with ideas for their own journeys.

## Data Availability

The datasets generated and analyzed during this study will not be made publicly available as per the research agreements created with each participating early learning and child care (ELCC) center.

## Appendix

Multimedia Appendix 1 Steering committee overview and author positionality statements.

Multimedia Appendix 2 Differences between PRO-ECO 1.0 pilot study and PRO-ECO 2.0 study.

## Abbreviations

5 R’s: 5 R’s of Indigenous Research (Respect, Relevance, Responsibility, Reciprocity, and Relationship)
AHSABC: Aboriginal Head Start Association of British Columbia
BCACCS: BC Aboriginal Child Care Society
CONSORT: Consolidated Standards of Reporting Trials
ECE: early childhood educator
ECEBC: Early Childhood Educators of British Columbia
ECPN: Early Childhood Pedagogy Network
ELCC: early learning and child care
iUBC: Innovation University of British Columbia
MNBC: Métis Nation British Columbia
OBM: observational behavior mapping
OCAP®: Ownership, Control, Access, Possession
PRO-ECO: Promoting Early Childhood Outside
SCT: social cognitive theory
SPIRIT: Standard Protocol Items: Recommendations for Interventional Trials
TOPO: Tool for Observing Play Outdoors
T-TRiPS: Teacher Tolerance of Risk in Play Scale
UBC: University of British Columbia
