# Supplementary material for: The 5 R’s of Indigenous Research as a Framework to Co-Design and Evaluate an Outdoor Play Program in Early Learning and Child Care Centers: Protocol for the Promoting Early Childhood Outside (PRO-ECO) 2.0 Wait-List Control Cluster Randomized Trial
Source: JMIR Res Protoc. 2025 Dec 12;14:e77956. doi: 10.2196/77956 (PMC12743237; doi:10.2196/77956)
Supplement: Multimedia Appendix 1 [file resprot_v14i1e77956_app1.docx]

# Multimedia Appendix 1: Steering committee overview and author positionality statements.

Table S1: PRO-ECO 2.0 Committee Members

| **Member** | **Organization/ University** |
| --- | --- |
| Stella Erasmus Johnson* | Knowledge Keeper, Vancouver Island University |
| Jean Lloyd* | Knowledge Keeper |
| Trudy Hill* | Aboriginal Head Start Association of BC |
| Kirsten Bevelander, Klara Schoenfeld & Angela Wrede | BC Aboriginal Child Care Society |
| Enid Elliot* | Camosun College/ Learning Outside Together Program |
| Emily Mlieczko* & Andrea Lemire* | Early Childhood Educators of BC |
| Kathleen Kummen | Early Childhood Pedagogy Network |
| Laranna Scott* & Trena Hebert | Métis Nation British Columbia |
| Ally Dai* | Family representative |
| Ashley Barrett* | Family representative |
| Emma Cottier* | Family representative |

* Steering Committee Members that also chose to be included as authors.

## Steering Committee Member Authors’ Positionality Statements

Knowledge Keeper Stella Johnson, nee Erasmus is Métis and Cree, was born in McLennan, Alberta, raised by her grandmother from age 5 and taught the Cree language. Auntie Stella’s father was Sam Erasmus, son of George Erasmus, son of William Erasmus, son of Peter Erasmus whose calling was as the translator of treaties. Peter Erasmus was married to Catherine ‘Kitty’ Budd, whose parents were William Hemings Cook and Kahaawoswamakan Agatha Cree. Her mother was Marguerite (Margaret) Noose-key-ah, whose parents were Joseph “Joe” Brilliant Noose-key-ah and Eliza Collin. Auntie Stella’s mother was Doris Mabel Becker, whose parents were Gordon Becker and Julie Ghostkeeper of Charlevrit, whose parents were Charles Ghostkeeper and Therese Cartier. Auntie Stella has been a Métis knowledge keeper at Vancouver Island University since 2012. She acknowledges the strength of outdoor opportunities for early learning and child care programs, and the importance of sharing Cree and Métis ways, culture, language, ways of living with children. Language and culture go hand in hand and being outdoors provides opportunity to teach new words, such as the names of plants, weather patterns, and counting. She supports her own grandchildren with their learning and has been inspired to do things with her great grandchildren. Auntie Stella enjoys being on the PRO-ECO 2.0 committee to learn and to introduce Indigenous perspectives. She has seen how children change when outdoors, and participate in more diverse and fulsome ways.

Knowledge Keeper Jean Lloyd is Métis and lives in Grand Forks, BC. She describes her participation in PRO-ECO 2.0 as reminiscent of her presence during the building, opening, and as an elder, caring for children in the local AHSABC facility. Witnessing children outdoors in an open space exploring the Earth Mother, is a great freedom and gift to us all. Behaviours melt away, grounding occurs, curiosity takes over, and a sense of wonder lands right before our eyes. Elder Jean regularly attends the facility benefiting from PRO-ECO 2.0. While the playground changes were going in, she saw a mother and her child carrying small plants to their new location, carefully placing them, the child returning carrying a small container of water to care for each plant, and then finding the water hose to water a greater area with a greater effect! Seeing the incredible transformation of the play areas, the excitement of staff and children, is a heart gift. Elder Jean smiles at the thought of the joy, healing, and delight the children, families, and communities experience as a result of the work of PRO-ECO 2.0, staff, the Steering Committee, and the many volunteers; all with incredible skills and knowledge to make all of this possible. Elder Jean sees the sense of pride and gratitude in the children, staff, community, and the Committee for the sum total of each person’s individual parts, as so much greater than we could know when the work began.

Trudy Hill is Turtle Clan from Oneida Nation of the Thames (Iroquois). She has worked with Aboriginal Head Start since 1997 and describes it as the first time she experienced celebrating Indigenous children and their families. Trudy believes in transparency and advocates for parents by giving them the information they need to make informed decisions. She supports research and partnerships that are grassroots lead and promotes data sovereignty. Trudy firmly believes in the Seventh Generation Principle, based on the Haudenosaunee teaching that decisions we make today should results in a sustainable world seven generations into the future.

Enid Elliot is a settler, an early childhood educator and a researcher. Recently retired from Camosun College and adjunct professor at University of Victoria she is a long-time advocate of outdoor play in early childhood education. Grateful for the gifts of land and inspired by the energy and curiosity of young children she participated in the development and writing of the LOT program.

Emily Mlieczko, Executive Director of ECEBC, represented ECEBC on the PRO-ECO steering committee. She is of settler descent and is an advocate for educators, families and children. Early Childhood Educators of BC (ECEBC), Social Research and Demonstration Corporation (SRDC) and BC Aboriginal Child Care Society (BCACCS) partnered to develop the Learning Outside Together (LOT) program that provided the professional learning and mentorship program component of PRO-ECO. The LOT program’s focus on Etuaptmumk (Two-Eyed Seeing) was a transformative experience for her and ECEBC. As a provincial organization whose mandate is to advance the quality and professionalism of early care and learning, this work has changed how the organization engages daily. Especially, deepening relationships with Indigenous partners, and the need to listen, respond and support the ongoing growth of both LOT and PRO-ECO. As a non-Indigenous organization working with Indigenous partners, we are learning to weave together two worldviews in authentic ways to better support educator’s practice. Two-Eyed Seeing has highlighted ECEBC’s obligations as a provincial organization at all levels and has been a motivating factor in the participation of the PRO-ECO project.

Andrea Lemire is a first-generation immigrant on her mother's side, while her father's ancestors immigrated to these lands thirteen generations ago. She is an uninvited guest of mixed settler descent who was employed as the Project Manager of the LOT program. Andrea came to this work with the Western worldview of Children's Rights and received lasting lessons from the Elders and Knowledge Keepers whose stories and wisdom shaped both LOT program itself and how we worked in the creation, implementation, and evaluation of the LOT program. She raises her hands in gratitude to these individuals and all the members of the LOT Steering Committee.

Laranna Scott is Métis, Danish and Russian and has worked in the Early Childhood and Child & Youth Care field in a variety of capacities for nearly 25 years. She has a passion for supporting the health and well-being of children and families, has a strong connection to the land and has witnessed the many benefits of sharing this with children.

Ashley Barrett is a First Nations member of the Treaty 8 Territory and joined the Steering Committee as a parent partner. She sought to become more involved in daughter’s education experience, including the school experience and support for the staff.

Emma Cottier is a dedicated and observant parent partner, whose unwavering belief in the power of nature-based learning stems from witnessing her son’s remarkable growth in a dynamic, outdoor preschool environment. She supports the transformative impact of unstructured and varied outdoor play and how it fosters resilience, creativity, and a profound connection to the natural world in young children.

Ally Rice is a non-Indigenous family partner living on the unceded territory of the Ktunaxa Nation, grateful that her children also grow and play on this land.
