# Supplementary material for: The 5 R’s of Indigenous Research as a Framework to Co-Design and Evaluate an Outdoor Play Program in Early Learning and Child Care Centers: Protocol for the Promoting Early Childhood Outside (PRO-ECO) 2.0 Wait-List Control Cluster Randomized Trial
Source: JMIR Res Protoc. 2025 Dec 12;14:e77956. doi: 10.2196/77956 (PMC12743237; doi:10.2196/77956)
Supplement: Multimedia Appendix 2 [file resprot_v14i1e77956_app2.docx]

# Multimedia Appendix 2: Differences between PRO-ECO 1.0 pilot study and PRO-ECO 2.0 study.

Table S2: Differences between the PRO-ECO 1.0 pilot and 2.0 studies, reflecting the 5 R’s

| **Component** | **PRO-ECO 1.0 pilot** | **PRO-ECO 2.0** |
| --- | --- | --- |
| Data ownership and stewardship | Outside Play Lab, UBC | - Ownership: ELCC centre communities - Stewardship: Outside Play Lab, UBC |
| ELCC centre inclusion criteria | ELCC centres within the YMCA Greater Vancouver region | ELCC centres throughout the province, prioritizing those serving Indigenous or equity-seeking families |
| Primary outcome | Play vs non-play, as measured through observational behaviour mapping | Bio-play, as measured through observational behaviour mapping. This measures children engagement with natural materials through the course of their play |
| Children’s outcomes and measurement methods | Surveys completed by ECEs about each child focused on children’s psychological and behavioural difficulties | Interviews with children on their well-being in their ELCC centres |
